# Structural Basis for GTP-Dependent Dimerization of Hydrogenase Maturation Factor HypB

**DOI:** 10.1371/journal.pone.0030547

**Published:** 2012-01-20

**Authors:** Kwok-Ho Chan, Ting Li, Ching-On Wong, Kam-Bo Wong

**Affiliations:** Centre for Protein Science and Crystallography, School of Life Sciences, The Chinese University of Hong Kong, Shatin, Hong Kong Special Administrative Region, The People's Republic of China; Institut Européen de Chimie et Biologie, France

## Abstract

Maturation of [NiFe]-hydrogenase requires the insertion of iron, cyanide and carbon monoxide, followed by nickel, to the catalytic core of the enzyme. Hydrogenase maturation factor HypB is a metal-binding GTPase that is essential for the nickel delivery to the hydrogenase. Here we report the crystal structure of *Archeoglobus fulgidus* HypB (AfHypB) in apo-form. We showed that AfHypB recognizes guanine nucleotide using Asp-194 on the G5 loop despite having a non-canonical NKxA G4-motif. Structural comparison with the GTPγS-bound *Methanocaldococcus jannaschii* HypB identifies conformational changes in the switch I region, which bring an invariant Asp-72 to form an intermolecular salt-bridge with another invariant residue Lys-148 upon GTP binding. Substitution of K148A abolished GTP-dependent dimerization of AfHypB, but had no significant effect on the guanine nucleotide binding and on the intrinsic GTPase activity. *In vivo* complementation study in *Escherichia coli* showed that the invariant lysine residue is required for *in vivo* maturation of hydrogenase. Taken together, our results suggest that GTP-dependent dimerization of HypB is essential for hydrogenase maturation. It is likely that a nickel ion is loaded to an extra metal binding site at the dimeric interface of GTP-bound HypB and transferred to the hydrogenase upon GTP hydrolysis.

## Introduction

Hydrogenases catalyze the inter-conversion of molecular hydrogen into protons and electrons. Hydrogenases can be categorized by coordination of their metallo-catalytic Fe core, namely, [FeFe]-hydrogenase, [NiFe]-hydrogenase and [Fe]-hydrogenase [1]. [NiFe]-hydrogenase, the most widely distributed hydrogenase, which contains a nickel and an iron ions coordinated by a network of thiol ligands from cysteine residues in the catalytic core of the large subunit. In addition, the Fe ion is chelated by two CN molecules and one CN molecule [1]. Formation of this complex catalytic core in the large subunit of [NiFe]-hydrogenase requires accessory proteins encoded by genes designated *hypA* to *hypF* in the *hyp* operon. The iron center is assembled before the insertion of nickel [2], [3], [4]. HypE and HypF are responsible for the synthesis of the CN ligands [5], [6], [7]. Synthesized CN ligands are transferred to HypC and HypD to form a Fe(CN)_2_ complex, which is then delivered to the hydrogenase large subunit precursor [8]. The source of the CO ligand is unknown but the insertion of Fe(CN)_2_CO should happen before the insertion of nickel [9], [10], [11]. After delivery of the Fe(CN)_2_CO complex to the large subunit precursor, nickel is transferred to the precursor with aid of protein HypA and HypB. The last step of catalytic core assembly involves protease cleavage, where an isozyme-specific protease cleaves the C-terminal tail of the large subunit [12].

The detailed process of nickel insertion facilitated by protein HypA and HypB is still unclear. Disruption of either genes *hypA* or *hypB* in various microorganisms resulted in hydrogenase deficiency that can be partially overcome by supplementation of nickel in the growing media [13], [14], [15], [16], [17], [18]. HypB is a metal-binding GTPase. It possesses an invariant CHx_n_C motif, which is capable to bind both zinc and nickel [19]. Mutagenesis study showed that this metal binding motif is essential for hydrogenase maturation [19], [20]. HypB belongs to the SIMIBI class GTPase with slow intrinsic GTP hydrolysis activity [21], [22], [23], [24]. Mutagenesis studies showed that GTP hydrolysis activity is required for hydrogenase maturation *in vivo* [15], [25], [26]. Size exclusion chromatography and cross-linking studies demonstrated that binding of GTP may trigger dimerization of HypB [21], [24], [27], which is further supported by the crystal structure of *Methanocaldococcus jannaschii* HypB (MjHypB) homo-dimer in complex with GTPγS [27]. Many GTPases, including HypB, undergo GTP-induced dimerization and in a recent review, Gasper *et al.* categorized them as “G proteins activated by nucleotide-dependent dimerization” [28]. Interestingly, a recent study showed that binding of nickel also triggers dimerization of HypB [29] suggesting that binding of metal and the oligomeric state are also related.

Here, we solved the crystal structure of *Archeoglobus fulgidus* HypB (AfHypB) (PDB: 2WSM) in its nucleotide-free apo-form. Structural comparison of the apo-form of AfHypB and the GTPγS-bound MjHypB (PDB: 2HF8) reveals a plausible GTP-dependent dimerization mechanism of HypB. Confirmed by mutagenesis study, an invariant lysine residue, which forms an intermolecular salt bridge across each copy of monomer in HypB dimer, was found to be required for GTP-dependent dimerization and *in vivo* hydrogenase maturation. How GTP-dependent dimerization of HypB plays a role in hydrogenase maturation is discussed.

## Results

### AfHypB undergoes GTP-dependent dimerization

**Figure 1:**
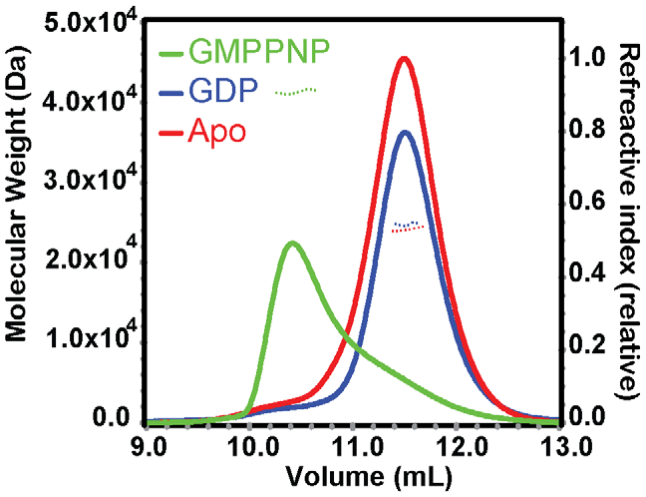
AfHypB undergoes GTP-dependent dimerization. Molecular weight of AfHypB in the presence of guanine nucleotides are determined by analytical size exclusion chromatography coupled with static light scattering. Both apo AfHypB (24.8 kDa) and GDP-bound AfHypB (23.6 kDa) remain as monomer (24.7 kDa). In contrast, AfHypB has an increased apparent molecular weight (41.0 kDa) in the presence of GMPPNP.

Previous study showed that GTPγ-S-bound HypB from *M. jannaschii* (MjHypB) exists as a dimer in the crystal structure [27]. Moreover, GTP-bound *E. coli* HypB (EcHypB) has a smaller elution volume than its GDP-bound state in size exclusion chromatography, suggesting that HypB has a tendency to dimerize in the presence of GTP. To test if AfHypB undergoes GTP-dependent dimerization, oligomeric state of AfHypB in the presence of GDP and GTP was studied by size exclusion chromatography coupled with static light scattering (SEC/LS) (Figure 1). The elution volume of both apo-form and GDP-bound AfHypB were 11.5 ml. The molecular mass, detected by SEC/LS, of apo-form and GDP-bound AfHypB was 24.8 and 23.6 kDa, respectively, which is consistent with the theoretical mass of monomeric AfHypB (24.7 kDa). These results suggest that the apo-form and the GDP-bound form of AfHypB exist as monomer in solution. On the other hand, the elution volume of GTP-bound AfHypB was significantly smaller (10.2 ml). The apparent molecular mass detected was 41.0 kDa, suggesting that AfHypB has a tendency to form dimer in the presence of GTP. Similar to EcHypB, our results suggest AfHypB undergoes GTP-dependent dimerization.

### Overall structure of AfHypB

**Figure 2:**
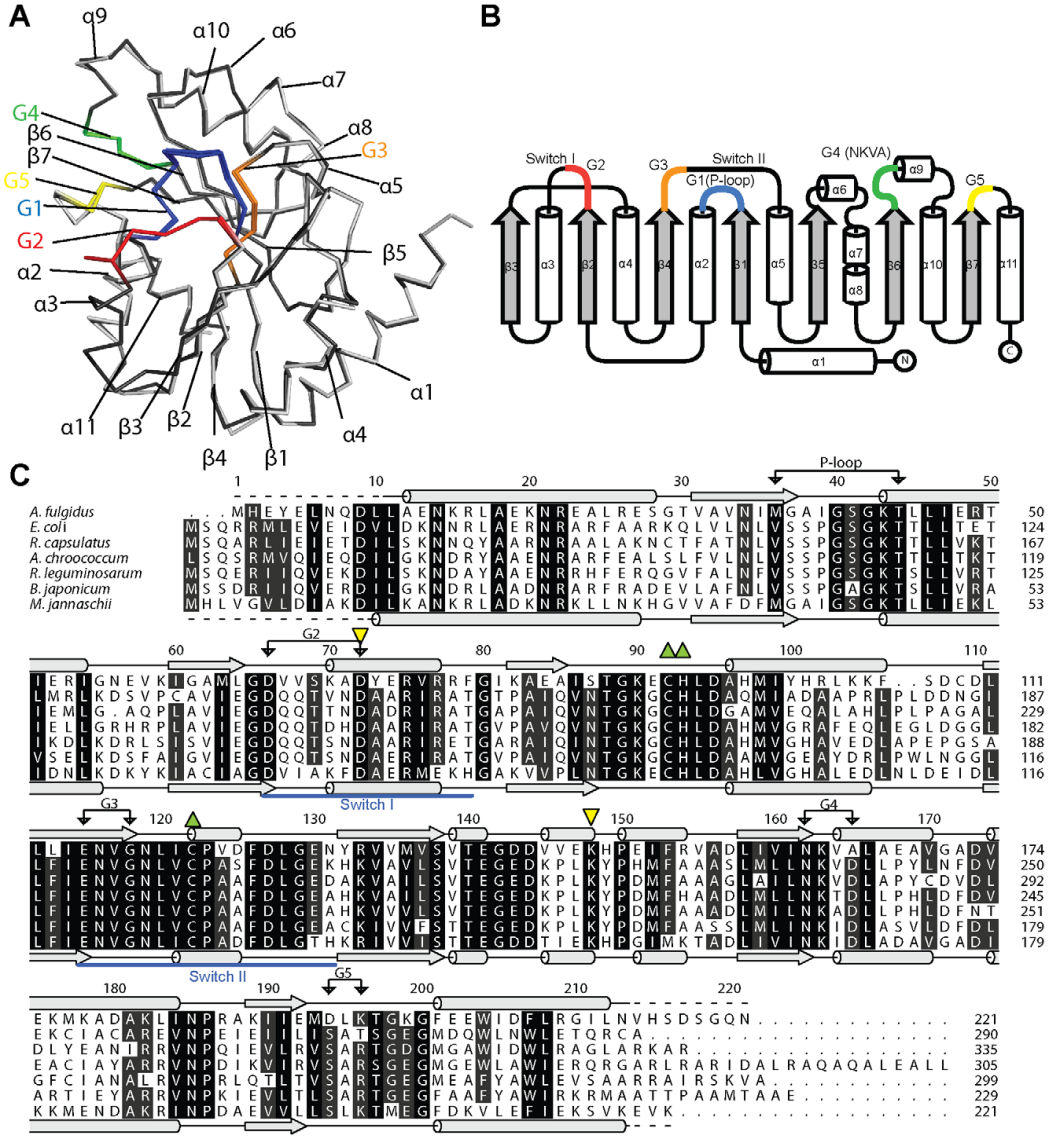
AfHypB is a SIMIBI class GTPase. (A) The two monomers found in the asymmetric unit of AfHypB are superimposable with Cα rmsd of 0.40 Å. G-motifs are annotated and high-lighted in colors. (B) HypB is a SIMIBI class GTPase and the secondary structure of AfHypB is illustrated in a schematic diagram. (C) Sequences of HypB from *A. fulgidus* (AfHypB), *M. jannaschii* (MjHypB) and five other species are aligned. The secondary structures of AfHypB and MjHypB are shown above and below the alignment, respectively. The invariant CHx_n_C-motif responsible for metal binding is indicated as green triangles. Asp-72 and Lys-148 (yellow triangles) are found to be absolutely conserved among all HypB.

The only structure of HypB available was the MjHypB in complex with GTPγ-S [27]. To better understand the structural mechanism of GTP-dependent dimerization, we have determined the crystal structure of HypB from *A. fulgidus* (AfHypB) in apo form to 2.3 Å. Each asymmetric unit contains 2 monomers of AfHypB (Figure 2A). The two monomers of AfHypB can be superimposable with each other with Cα rmsd of 0.40 Å. AfHypB belongs to the SIMIBI class of GTPase and adopts an α/β fold with a seven-stranded beta-sheet sandwiched by 11 α-helices (Figure 2B). Electron density of residues 67–82, corresponding to the switch I region, of chain B are missing (Table 1), indicating that these residues are not ordered. In contrast, we found that symmetry-related AfHypB molecules may restrict the mobility of switch I of chain A, and hence, these residues are well defined in chain A. Besides, the side-chain sulphur atoms of two cysteine residues, Cys-92 and Cys-122, which are part of the CHx_n_C motif, of apo-AfHypB structure are in 2 Å distance, suggesting the formation of a disulphide bridge between these two residues.

**Table 1:** Crystallographic Data and Refinement Statistics.

**Diffraction data statistics**	
Space group	P2_1_2_1_2
*a, b*, and *c* (Å)	72.49, 82.32, 68.66
Wavelength (Å)	1.5418
Resolution (Å)	42.76–2.3 (2.42–2.3)
R_merge_ (%)	10.7 (25.0)
Mean I/σI	20.0 (5.4)
Completeness (%)	100 (100)
Multiplicity	12.5 (7.9)
No. of reflections observed	235073 (21277)
No. of reflections unique	18861 (2705)
**Refinement**	
R_work_/R_free_ (%)	22.3/27.5
**No. of atoms**	
Protein	3018
Water	138
Chloride	1
**r.m.s.d. from ideal values**	
Bond lengths (Å)	0.005
Bond angles (°)	0.829
**Ramachandran plot analysis**	
Most favored region	94.2%
Additionally allowed region	5.8%
Other	0%
**Unmodeled residues**	
Chain A	1–10, 214–221
Chain B	1–8, 67–82, 212–221

*Values in parenthesis are for the highest resolution shell.*

### AfHypB can recognize guanine nucleotide despite having a non-canonical G4 motif

**Figure 3:**
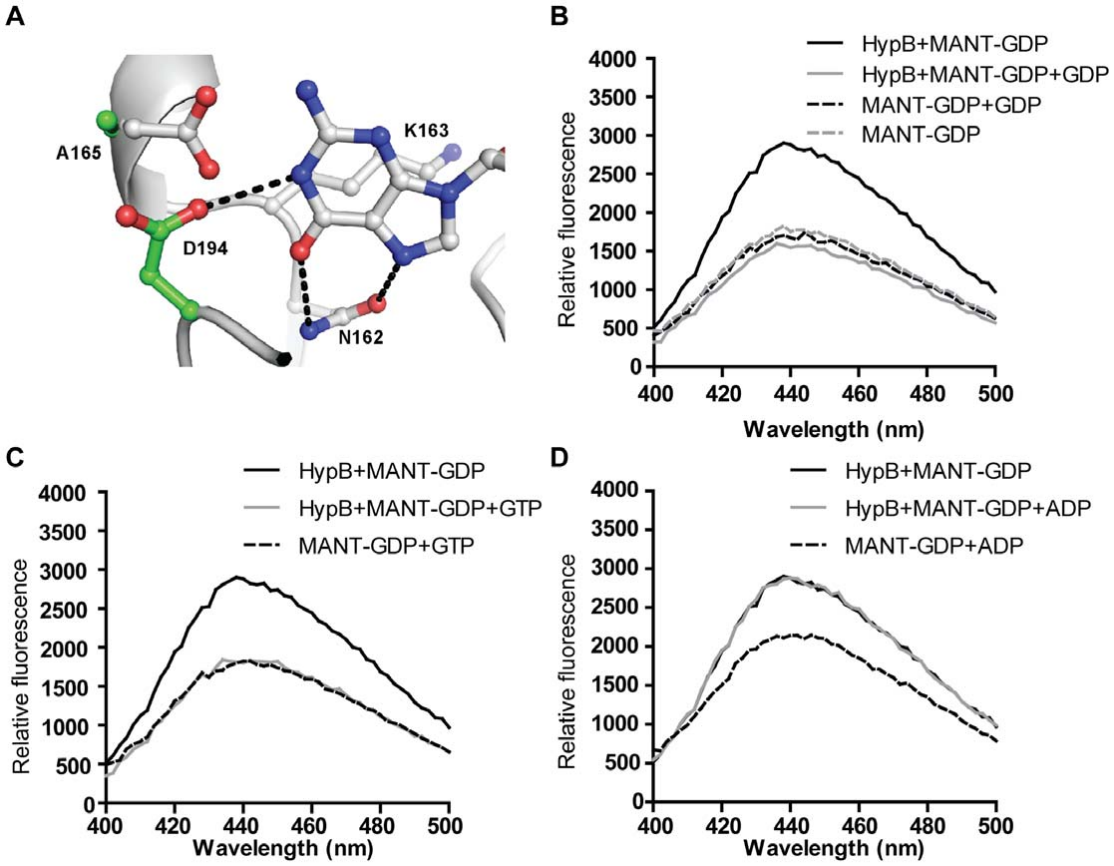
AfHypB can recognizes guanine nucleotide despite having a non-canonical NKxA G4-motif. (A) In MjHypB, the aspartate residue of the NKxD motif serves as a hydrogen acceptor that form specific hydrogen bonds with the guanine N1 and N2 atoms. In AfHypB (green), the aspartate residue in the canonical NKxD G4-motif is replaced by an alanine residue (Ala-165). The role of a hydrogen acceptor is fulfilled by a nearby Asp-194 on the G5 loop that can form hydrogen bond to the N1 atom of the guanine base. (B–C) AfHypB specifically binds GDP and GTP but not ADP. Binding of MANT-GDP (grey dashed line) to AfHypB resulted in an increase in fluorescence at wavelength 400–500 nm with excitation at 290 nm (black solid lines). Addition of excess GDP or GTP (grey solid lines) resulted in significant decreases of fluorescence, indicating both GDP and GTP could competitively displace MANT-GDP from AfHypB. (D) On the other hand, addition of excess ADP (grey solid line) resulted in no significant changes in fluorescence, indicating that AfHypB binds specifically to guanine nucleotide but not to adenine nucleotide.

Sequence of AfHypB was compared with HypB from *M. jannaschii* and five other species (Figure 2C). All G-motifs for guanine nucleotide recognition are identified with the exception of the G4 NKxD motif. In many GTPase including MjHypB, the conserved aspartate residue in the NKxD motif recognizes the guanine nucleotide by forming two hydrogen bonds with the N1 and N2 atoms on the guanine base. In AfHypB, the conserved Asp residue in the NKxD motif is replaced by an Ala residue. We have superimposed the structures of AfHypB and MjHypB, and found that there is a nearby Asp-194 on the G5 loop, away from the G4 motif, that can form a hydrogen bond to the N1 atom of the guanine nucleotide (Figure 3A). This observation suggests that the role of the conserved Asp in NKxD can be replaced by this Asp-194. We tested the ability of AfHypB to distinguish GDP and ADP by titration experiment using a fluorescent 2′-(or-3′)-*O*-(*N*-methylanthraniloyl)-GDP (MANT-GDP) analog. When 1 µM MANT-GDP was added to 1 µM apo-AfHypB, the fluorescence emission at wavelength 440–450 nm was increased (Figure 3B). Addition of 200 µM GDP or GTP resulted in a drop in the emitted fluorescence, suggesting that the bound MANT-GDP can be displaced by the addition of excess guanine nucleotides (Figure 3B & 3C). In contrast, no significant change in fluorescence was observed when 200 µM ADP was added (Figure 3D), suggesting that ADP cannot displace the bound MANT-GDP. Taken together, our results suggested that AfHypB binds guanine nucleotide specifically over adenosine nucleotide despite having a non-canonical G4-motif.

### Structural difference between the apo-form and GTPγS-bound form suggests a mechanism of GTP-dependent dimerization for HypB

**Figure 4:**
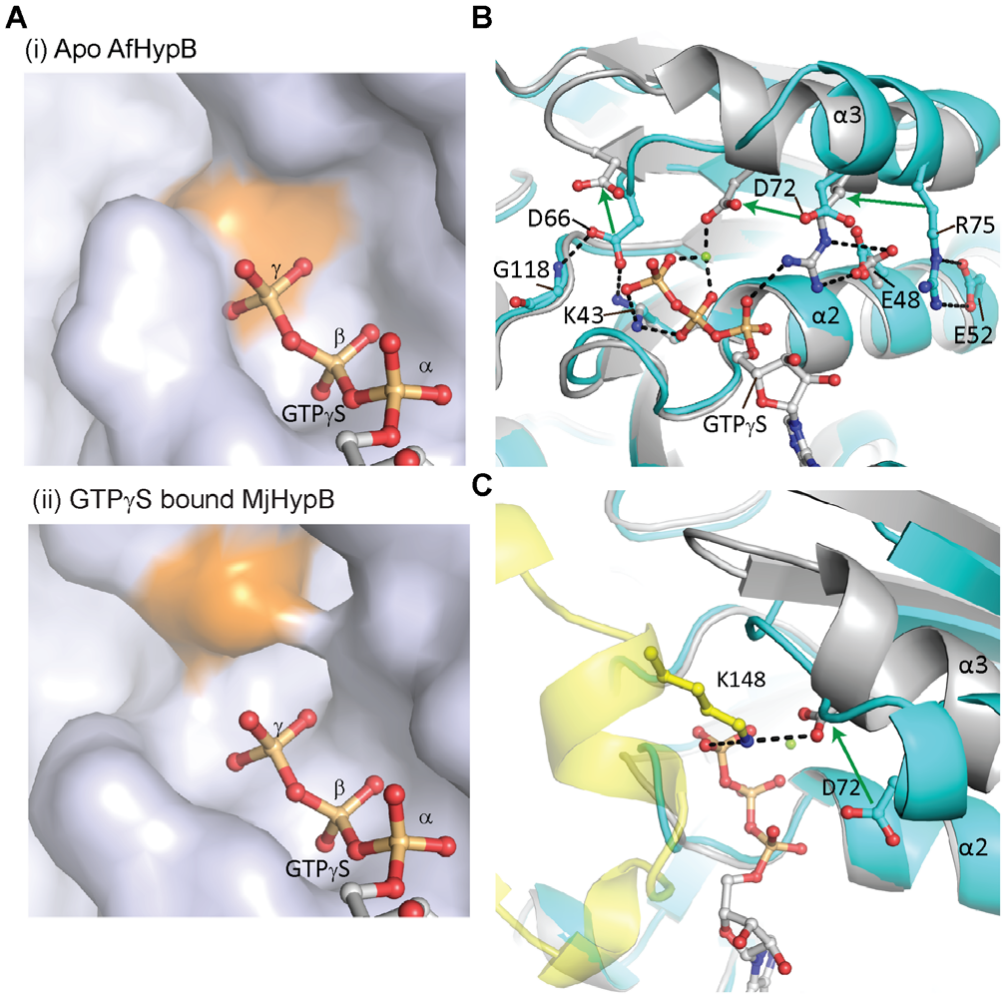
Binding of GTP to HypB causes conformational changes leading to HypB dimerization. (A) Surface representation of (i) apo-AfHypB (ii) GTPγS-bound MjHypB. A GTP molecule is modeled into the apo-AfHypB for comparison. In apo-AfHypB, the invariant Asp-66 occupies the γ-phosphate binding pocket. Therefore, it is not possible for HypB to accommodate a GTP molecule in such conformation. The surface of the invariant Asp is colored in orange. (B) Binding of GTPγS causes conformational changes in the switch I loop and helix-3. The movements of the conserved residues Asp-66, Asp-72, and Arg-75 are indicated by arrows. Apo-AfHypB and GTPγS-MjHypB are colored cyan and white respectively. (C) Binding of GTP causes swinging movement of helix-3 from the apo-conformation (cyan) to the GTP-bound state conformation (white). It causes Asp-72 to move by 4.9 Å to the dimeric interface. This allows Asp-72 to form a salt bridge with Lys-148 on the opposite chain (yellow). In apo-form conformation of HypB, the switch I loop blocks the site for Lys 148 insertion. All residues are numbered with reference to the sequence of AfHypB.

We have tried, but unfortunately failed to obtain crystals of AfHypB in complex with GDP or GTP analogs. To reveal conformational changes upon binding of GTP to apo-HypB, the structure of apo-AfHypB is compared with that of the GTPγS-bound MjHypB. AfHypB and MjHypB share 52% sequence identity, and the two crystal structures of apo-AfHypB and GTPγS-bound MjHypB are superimposable with Cα rmsd of 1.56 Å (chain A of apo-AfHypB against chain A of GTPγS-bound MjHypB) (Figure S1A). Modeling suggests that the binding pocket in apo-HypB can accommodate a GDP but is too small to accommodate a GTP as the γ-phosphate will clash with an invariant aspartate residue (Asp-66 in AfHypB) on the G2 loop (Figure 4A). As indicated by the large values of Cα displacement (Figure S1B), conformational changes are most evident in the switch I region (residues 65–81) consisting of helix-3 (residues 70–77) and the G2 loop (residues 65–69). Three conserved residues, Asp-66, Asp-72 and Arg-75 (Figure S1C), are involved in the observed conformational changes upon GTP binding. First, in the apo-form, Asp-66 on the G2 loop is anchored to Gly-118 and Lys-43 by two hydrogen bonds, which are broken in the GTPγS-bound MjHypB (Figure 4B). These contacts are observed in both chain A and chain B in the crystal structure of apo-AfHypB. These two hydrogen bonds hold the G2 loop closer to nucleotide binding site in the apo-form, resulted in a smaller guanine nucleotide-binding pocket that clashes with the γ-phosphate group of GTP (Figure 4A). We anticipate that binding of GTP breaks the hydrogen bonds among Lys-43, Gly-118 and Asp-66, allowing the switch I to swing away from the nucleotide binding site. Such motion results in a more open conformation for the accommodation of the γ-phosphate group of GTP, and the insertion of Lys-148 from the opposite chain for dimerization of HypB (Figure 4C). Second, Arg-75 of helix-3 forms salt bridge with Glu-52 of helix-2 in the apo-form. In the GTPγS-bound form, helix-3 turns ∼30°, bringing Arg-75 to form salt bridge with α-phosphate of GTPγS and Glu-48. Such conformational changes also bring Asp-72 in a position to interact with the bound magnesium, and to form an intermolecular salt bridge with Lys-148 in GTP-bound form.

### Lys-148 is essential for GTP-dependent dimerization

**Figure 5:**
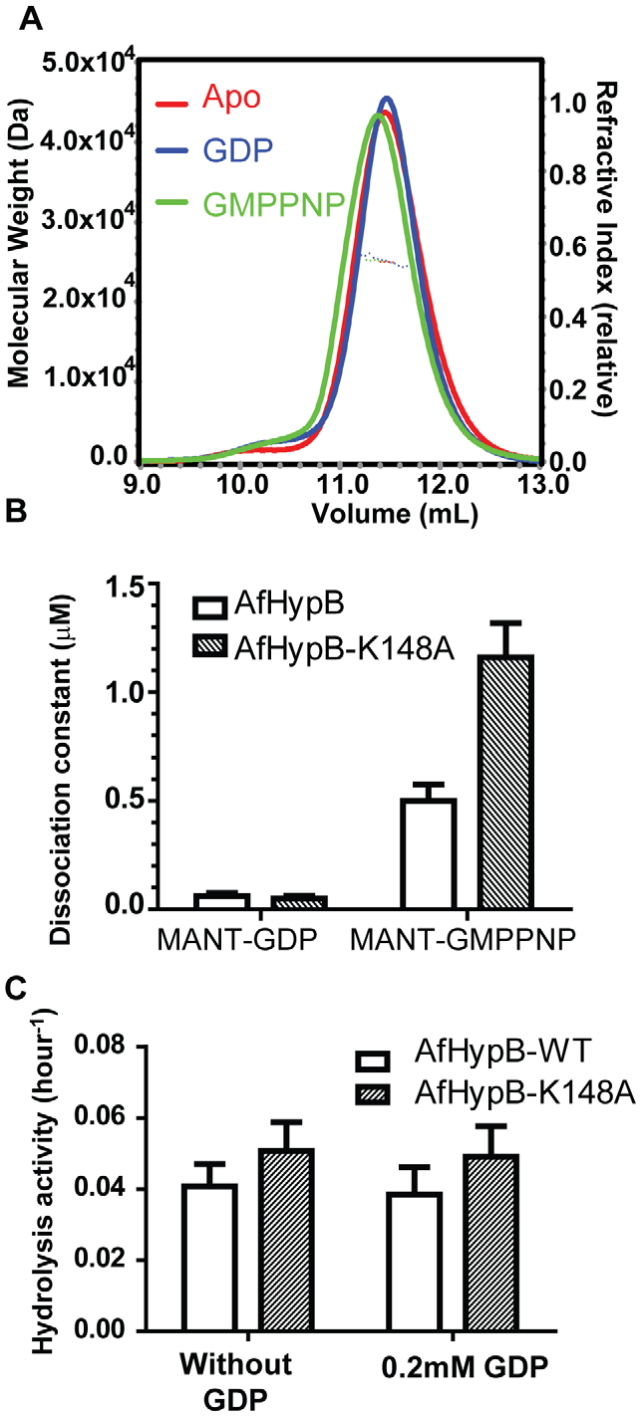
Substitutions of K148A disrupted GTP-dependent dimerization of AfHypB, but has no significant effect on guanine nucleotide binding and GTPase activity. (A) Molecular weight of AfHypB K148A variant in the presence of 5 mM GDP (blue), GMPPNP (green) or without nucleotides (red) were determined by SEC/LS. (B) The dissociation constants of AfHypB and the K148A variants for binding MANT-GDP and MANT-GMPPNP. (C) GTP hydrolysis rate in the presence of AfHypB or K148A mutant were determined as free phosphate release per hour (left). 0.2 mM of purified protein was mixed with 2 mM GTP. The GTP hydrolysis rates of wild-type and K148A AfHypB were found to be 0.041±0.006 hr^−1^ and 0.051±0.008 hr^−1^. Error bars indicate the standard deviation of hydrolysis rate over three independent experiments. The same experiment was repeated with protein pre-incubated with equimolar of GDP (right). The GTP hydrolysis rates of wild-type and K148A AfHypB pre-incubated with equimolar of GDP were found to be 0.039±0.008 hr^−1^ and 0.049±0.008 hr^−1^. Pre-incubation of GDP did not show significant effect to GTP hydrolysis activity of AfHypB or the K148A variant.

Structural comparison between apo-AfHypB and GTPγS-bound MjHypB suggests that conformational changes induced by GTP binding bring Asp-72 to form an intermolecular salt-bridge with Lys-148. As both Asp-72 and Lys-148 are invariant residues in HypB, we hypothesized that this salt bridge is essential for the GTP-dependent dimerization of HypB. To test our hypothesis, Lys-148 of AfHypB was substituted by alanine to create AfHypB-K148A and its oligomeric state was analyzed by SEC/LS (figure 5A). In contrast to the wild-type AfHypB, addition of the GTP analog, GMPPNP, did not result in changes in the elution volume in SEC/LS analysis. The detected molecular mass of K148A was ∼25 kDa, suggesting that K148A exists as a monomer in the absence or in the presence of GDP and GMPPNP. The K148A variant failed to undergo GTP-dependent dimerization. Our results are consistent with the conclusion that the intermolecular salt bridge between Lys-148 and Asp-72 is important for GTP-dependent dimerization.

### K148A substitution did not affect nucleotide binding and GTP hydrolysis activity

Next, we questioned if GTP-dependent dimerization is essential for the binding of guanine nucleotide. The binding of MANT-guanine nucleotide to wild-type and variants of AfHypB was followed by fluorescence changes at wavelength 450 nm (Figure S2). The dissociation constants of wild-type and K148A for MANT-GDP were 0.06±0.01 µM and 0.05±0.01 µM respectively and that for MANT-GMPPNP were 0.50±0.08 µM and 1.2±0.2 µM respectively (Figure 5B). That K148A AfHypB variant can bind guanine nucleotide suggests that nucleotide binding does not require dimerization.

To test if GTP-dependent dimerization affects the enzymatic activity of HypB, we measured the GTP hydrolysis activity for wild-type and K148A AfHypB by incubating 200 µM of HypB with 2 mM of GTP. (Figure S3). The hydrolysis activity was not affected by pre-incubation of HypB with 200 µM of GDP (Figure 5C), suggesting that the assay measured multiple turnover rates of GTP hydrolysis rather than the dissociation rates of the product GDP. The activity for wild type and K148A variant were 0.041±0.006 hr^−1^ and 0.051±0.008 hr^−1^ respectively, showing that K148A substitution did not affect the activity significantly.

### The conserved lysine residue is required for hydrogenase maturation in *E. coli*

**Figure 6:**
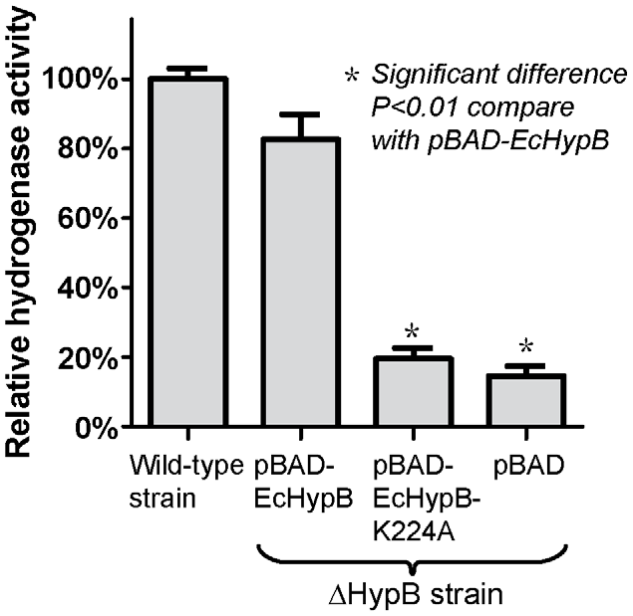
Mutation of the invariant lysine residue of *E. coli* HypB blocked *in vivo* hydrogenase maturation. The Δ*hypB* strain was transformed with the plasmids encoding wild-type EcHypB(WT), the K224A mutant, or the empty vector control pBAD. The hydrogenase activity of the lysates of these Δ*hypB* strains was compared relative to that of the wild type E. coli strain.

We have shown that removal of the positive charge at the conserved residue Lys-148 abolishes GTP-dependent dimerization without affecting the guanine-nucleotide binding and the intrinsic activity of HypB. Next, we asked if this conserved lysine residue is essential for the *in vivo* hydrogenase maturation. To address this question, the ability of the HypB variant to complement hydrogenase maturation in Δ*hypB E. coli* was tested. Sequence alignment suggests that Lys-224 in *E. coli* HypB (EcHypB) corresponds to Lys-148 in AfHypB (Figure 2C). We have cloned wild-type EcHypB and EcHypB-K224A constructs in pBAD plasmid vector. The hydrogenase activity measured for the wild-type parental *E. coli* strain was 302±9 nmol mg^−1^ min^−1^ (Figure 6). Transforming the Δ*hypB* strain with the wild-type pBAD-EcHypB plasmid recovered 83±7% of the activity observed in the wild type strain *E. coli*, suggesting that the expression of wild-type HypB complemented the loss of *hypB* gene in the Δ*hypB* strain genome. In contrast, transforming the Δ*hypB* strain with the pBAD-EcHypB-K224A plasmid only recovered 20±3% hydrogenase activity, which is similar to the activity recovered with the empty vector control (15±3%). Our results suggested that K224A could not complement the loss of *hypB* gene, indicating that this conserved lysine residue is essential for hydrogenase maturation.

## Discussion

Here we reported the crystal structure of apo-AfHypB. This apo-form conformation is reminiscent of that observed in Ras-like G-proteins in complex with guanine nucleotide exchange factors [30], [31], [32]. In the case of Ras-like G-proteins, nucleotide exchange is enhanced by deforming the nucleotide-binding pocket caused the conformational change upon binding of guanine nucleotide exchange factors. In this study, we showed that AfHypB pre-loaded with GDP can readily exchange with GTP without the help of exchange factors in the nucleotide displacement experiment (Figure 3C). The crystal structure of apo-AfHypB reported here may represent the intermediate state between the GDP- and GTP-bound form. The salt-bridge between Asp-66 in the switch I region and Lys-43 in the P-loop is likely stabilizing this intermediate “nucleotide-free” conformation of HypB that allows nucleotide exchange without the aid of other exchanges factors. However, it is not known if this intermediate conformation of HypB can be further stabilized by binding to other protein factors.

Consistent with previous observations [21], [23], [24], we showed that AfHypB undergoes GTP-dependent dimerization. Structural comparison of the apo-form and GTPγS-bound form of HypB reveals major conformational changes in the G2 loop and in helix-3, which turns ∼30° bringing Asp-72 to form an intermolecular salt-bridge with Lys-148 on the opposite chain. These two residues are absolutely conserved in all HypB, suggesting an indispensable role of these residues. Substituting the Lys-148 with alanine abolished GTP-dependent dimerization of HypB, suggesting the salt-bridge between Asp-72 and Lys-148 is essential for dimer formation. We further showed that dimerization did not affect nucleotide binding affinity and the GTP hydrolysis rate of HypB. Our results suggest that HypB can bind GTP in the monomeric state, and the induced conformational changes promote dimerization of HypB via the formation of the intermolecular salt-bridge between Asp-72 and Lys-148. The invariant Lys-148 serves as a probe for the binding of GTP that triggers the dimerization of HypB. Interestingly, substitution of this invariant lysine residue with alanine in EcHypB abolishes the ability of HypB to complement hydrogenase maturation in Δ*hypB E. coli* strain. Consistent with our observation, Cai *et al.* demonstrated that substitution of two hydrophobic residues (Leu-242, Leu-246) located at the dimeric interface by alanine disrupted dimerization of EcHypB, and greatly reduced the recovered hydrogenase activity in an *in vivo* hydrogenase maturation assay [33]. Taken together, our results suggest that maturation of hydrogenase involves GTP-dependent dimerization of HypB.

In this study, the GTP hydrolysis rate of AfHypB is very low, which is consistent with previous studies of HypB from other species [21], [22], [23], [24]. This observation suggests that other factors must involve in activating the catalysis of HypB. Gasper *et al.* suggested that hydrolysis reaction of these G-proteins, which undergo GTP-dependent dimerization, is coupled to interacting with the effectors and/or other GTPase co-regulators [28]. There is no experimental evidence demonstrating the role of effectors or co-regulators in activating GTP hydrolysis of HypB. It is likely that activation of HypB requires binding to its effector, hydrogenase. Moreover, it has been showed that nickel insertion involved both HypA and HypB [13], [14], [15], [16], [17], [18], [34]. From this point of view, it is possible that HypA may serve as a co-regulator in activating the GTP hydrolysis by HypB.

**Figure 7:**
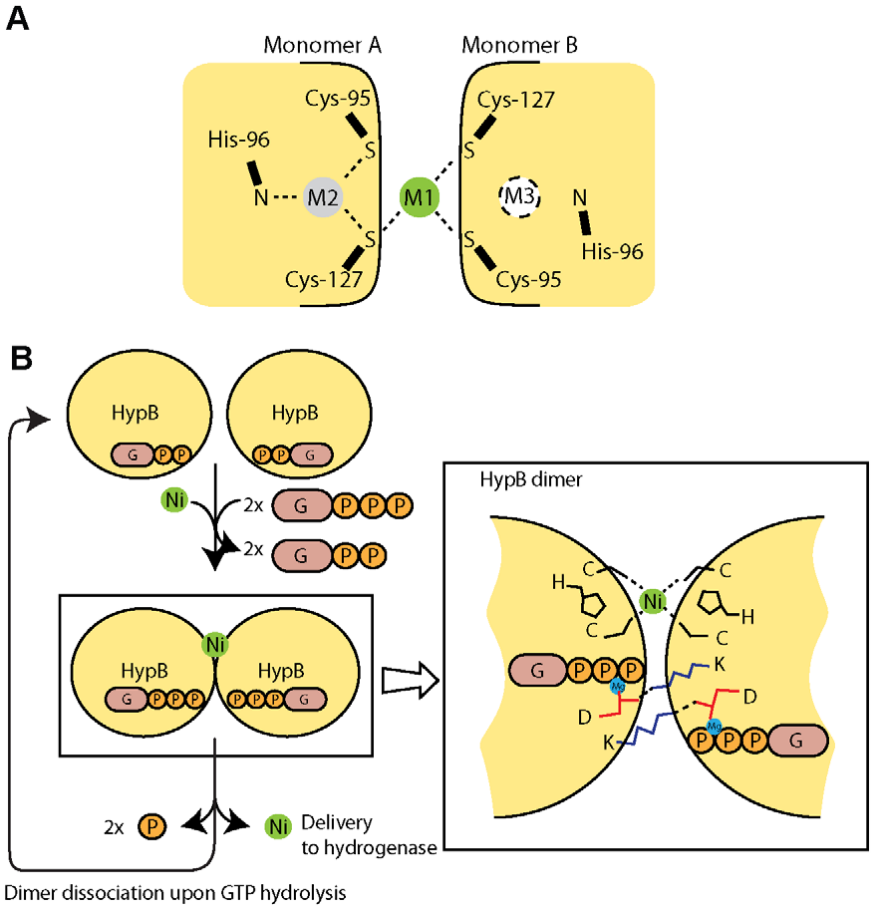
Dimerization of HypB allows the formation of an extra metal binding site in the dimeric interface. (A) Schematic diagram of metal binding sites in the crystal structure of dimeric MjHypB. Two zinc metal atoms were found in sites M1 and M2. M3 is the mirror site of M2 which presumably exists in monomeric HypB. Note that the M1 is only formed after GTP-dependent dimerization of HypB. (B) A model on how GTP-dependent dimerization may play a role in hydrogenase maturation. Upon binding of GTP, conformational changes of HypB lead to the formation of HypB dimer stabilized by the intermolecular salt bridge via the invariant lysine and aspartate residues (Lys-148 and Asp-72 in AfHypB). Formation of HypB dimer provides an extra metal binding site situated at the dimeric interface. Upon GTP hydrolysis, HypB dimer dissociates and the bound nickel is delivered to the hydrogenase.

How GTP-dependent dimerization plays a role in hydrogenase maturation? One possibility is that dimerization of HypB creates an extra metal binding site for nickel. It was observed that in the crystal structure of MjHypB-GTPγS complex, two zinc atoms are bound by three invariant residues Cys-95, His-96 and Cys-127 (we call these residues the CHx_n_C-motif) situated at the dimeric interface of HypB (Figure 7A) [27]. The CHx_n_C-motif has been shown by mutagenesis studies to be essential for metal binding and hydrogenase maturation [1], [19], [20]. Gasper *et al.* pointed out that zinc atoms bound in site M2 (or its mirror site M3) in MjHypB dimer via the CHx_n_C motif could be an artifact in protein crystal preparation and these sites may bind nickel in native HypB dimer [27]. Upon GTP-dependent dimerization, Cys-95 and Cys-127 on each monomer come in close proximity and hence forming a new site M1. We argue that this extra metal binding site at the HypB dimeric interface is likely to bind nickel instead of zinc in the native nickel delivery complex. This suggestion is supported by a recent work of Sydor *et al.*, which demonstrated that *H. pylori* HypB dimerizes in the presence of nickel and the nickel-dependent dimerization requires the CHx_n_C motif [29]. It is likely that nickel binds to the M1 site at the interface and the extra interactions formed promote the formation of HypB dimer.

Putting these together, a nickel delivery mechanism via GTP-dependent dimerization of HypB is proposed (Figure 7B). GTP-dependent dimerization facilitates nickel delivery to hydrogenase by loading nickel to the metal-binding site at the dimeric interface. Upon binding of GTP, the invariant lysine and aspartate residues (K148 and D72 in AfHypB) at the dimeric interface form two intermolecular salt bridges that bring the two copies of HypB monomer together. HypB dimerizes and the extra metal binding site M1 formed is loaded with nickel. The mechanism of nickel loading is currently not known, but another nickel chaperone HypA may play a role here. Although nickel contributes to the stabilization of HypB dimer as observed by Sydor *et al.* [29], it is expected at physiological conditions where both nickel and HypB concentrations are much lower, dimerization of HypB would be still GTP-dependent. GTP hydrolysis triggers HypB dimer dissociation and the bound nickel is released and transferred to the hydrogenase large subunit precursor.

The role of zinc in HypB dimerization and nickel delivery is unclear. Interestingly, the M2 site in the monomeric HypB has a higher affinity for zinc than nickel, suggesting that the site could be occupied by zinc in the monomeric state [19]. Zinc binding as a structural role has been observed in other related proteins. For example, a similar metal-binding CxH motif is present in UreG, a homolog of HypB that plays a role in urease maturation, where zinc binding stabilizes the unstructured region near this site [35]. Besides, HypA also binds zinc with a zinc-finger like Cys_4_ motif that stabilizes the structure of the zinc binding domain [36], [37]. Zinc binding in HypB may serve a similar structural role to maintain the conformation of the binding site. However, whether zinc is present on HypB in its nickel loaded GTP-bound dimer form is not known as HypB dimer in complex with both zinc and nickel is not observed yet.

## Materials and Methods

### Recombinant Plasmid Construction

The *hypB* genes were amplified by PCR using *A. fulgidus* and *E. coli* genomic DNA as templates, and were cloned into the expression vector pRSETA-His-SUMO (an in-house expression plasmid based on pRSETA (Invitrogen) with poly-His and SUMO-protein fusion tags at the N-terminus) and pBAD-A (Invitrogen) to create pRSET-His-SUMO-AfHypB and pBAD-EcHypB. Both constructs were verified by DNA sequencing. Primers used for PCR are listed in Table S1.

### HypB mutant construction by site-directed Mutagenesis

Variants of AfHypB and EcHypB were constructed by QuikChange mutagenesis kit from Stratagene on wild type HypB constructs pRSET-His-SUMO-AfHypB and pBAD-EcHypB to generate pRSET-His-SUMO-AfHypB-K148A and pBAD-EcHypB-K224A respectively. Mutagenesis was performed according to the manufacturer's instruction with the following modifications - Phusion (Finnzymes) was used to replace Pfu DNA polymerase and the concentration of template plasmid was increased from 10 ng to 100 ng. Mutants created and the primers used are listed in Table S1. All constructs were verified by DNA sequencing.

### Protein Expression and purification

To express AfHypB, the pRSET-His-SUMO-AfHypB was transformed to *E. coli* BL21 (DE3) pLysS host. The bacterial cells were grown in LB medium supplemented with 100 µg/mL ampicillin. Protein expression was induced by addition of 0.4 mM isopropyl-D-1-thiogalactopyranoside when OD600 reached 0.8. After 4 hours, the cells were harvested by centrifugation at 5,000× g, 15 min, 4°C. Cell pellets were stored in −20°C freezer before use.

Cell pellet expressing His-SUMO-AfHypB from 1 L culture was resuspended in 20 mL of lysis buffer (20 mM Tris-HCl pH 7.5 containing 500 mM NaCl, 50 mM imidazole and 1 mM TCEP), and lysed by sonication. Cell debris was removed by centrifugation at 12000× g for 30 min, and the supernatant was loaded onto a 5 mL HiTrap™ IMAC Column (GE Healthcare) charged with Ni (II) ion. Column was pre-equilibrated with the lysis buffer. After extensive washing with the lysis buffer, His-tagged protein was eluted with 300 mM imidazole in lysis buffer. The His-SUMO tag was cleaved by addition of 0.5 mg of His-tagged SUMO protease per 1 L of culture. After dialysis in 20 mM Tris-HCl pH 7.5 containing 200 mM NaCl and 1 mM TCEP at 4°C overnight, the fusion tag was removed by a second round of nickel affinity chromatography. The IMAC column was washed with 20 mM Tris-HCl pH 7.5, 0.2 M NaCl, 50 mM imidazole and 1 mM TCEP, and the AfHypB protein was in the flow-through and washing fractions. Protein sample was concentrated to 10 mg/mL, and loaded onto a HiLoad 26/60 Superdex 75 column equilibrated with 50 mM Tris-HCl buffer pH 7.5, 0.2 M NaCl and 1 mM TCEP. Eluted fractions were analyzed by SDS-PAGE. Purified AfHypB was eluted at elution volume of ∼165 ml. Apo-AfHypB was prepared by extensive dialysis in 50 mM Tris-HCl buffer pH 7.5, 0.2 M NaCl, 1 mM TCEP and 5 mM EDTA to remove the bound magnesium and nucleotide. We showed that EDTA can remove bound MANT-GDP from AfHypB (Figure S4). After removal of EDTA by dialysis, the protein sample was frozen in liquid nitrogen, and stored in −80°C freezer before use.

### Crystallization and Data Collection

Protein sample of AfHypB was prepared in 20 mM Tris-HCl buffer, pH 7.8, containing 200 mM NaCl, 5 mM MgCl_2_, and 5 mM TCEP and concentrated to 10–15 mg/mL. Crystals for diffraction data collection were grown in 8% PEG 4000, 0.1 M sodium acetate buffer at pH 4.6 using hanging-drop-vapor-diffusion method. Cryo-protection was achieved by soaking the crystals in mother liquor with 20% (v/v) (±)-2-Methyl-2,4-pentanediol. Diffraction data were collected to 2.3 Å resolution using an R-AXIS IV++ IP detector and Cu Kα X-rays generated by a Rigaku MicroMax-007 rotating anode generator, and were integrated, merged, and scaled using the programs MOSFLM [38], SCALA [39] and TRUNCATE [40] in the CCP4 suite [41]. The phase was solved by molecular replacement using the structure of GTPγS-bound HypB from *M. jannaschii* (PDB code: 2HF8) as template with the program MOLREP [42]. The model was built interactively using the program COOT [43], and refined by the program REFMAC [44]. There are two molecules of AfHypB in the asymmetric unit. No restraints on the non-crystallographic symmetry were imposed during refinement. Data collection and processing statistics are given in Table 1. Simulated annealing omit map was generated to show the electron density of the refined structure (figure S5). All figures of protein structures in this work were generated using the program PyMol (http://www.pymol.org).

### GTP-dependent dimerization study

The molecular weight of AfHypB was determined using size-exclusion chromatography (SEC) coupled with multiple-angle light scattering (SEC/LS). Protein samples (100 µL at 3 mg/mL) in the absence or in the presence of 1 mM GDP or GMPPNP were loaded onto a Superdex-75 HR10/30 column (GE Healthcare) pre-equilibrated with 50 mM Tris pH 7.5, 0.1 M NaCl, 5 mM MgCl_2_ and 1 mM TCEP, and eluted at room temperature at a flow rate of 0.5 mL/min. The column was connected downstream to a multiple-angle laser light scattering Mini-DAWN light scattering detector (Wyatt Technology). Data were analyzed using Astra version 5.3.4.18.

### Nucleotide binding affinity assay

The affinity of AfHypB towards guanine nucleotide was determined using fluorescence resonance energy transfer (FRET) between the tryptophan residues and the bound 2′-(or-3′)-*O*-(*N*-methylanthraniloyl) (MANT) nucleotide analogue. 0.05 µM MANT-GDP or 1.33 µM MANT-GMPPNP was incubated with 10 nM - 13 µM of AfHypB or AfHypB-K148A variant in 50 mM Tris pH 7.5, 0.2 M NaCl and 5 mM MgCl_2_ buffer. After 30 minutes incubation at 4°C. Emission spectra were collected using a Tecan Inifinite 200 Pro plate reader with excitation wavelength at 290 nm. Dissociation constant (K_d_) of HypB towards nucleotide was determined as described by Ahmadian *et al.* [45]. Data fitting was performed using the program Prism (GraphPad Software, Inc.).

### GTPase activity determination

0.2 mM of AfHypB and AfHypB-K148A was incubated with 2 mM of GTP at 37°C and the release of free phosphate of the mixture was monitored hourly by determining the free phosphate concentration in the sample using the ammonium molybdate method as described by Gawronski and Benson, 2004 [46].

### Hydrogenase activity assay

Hydrogenase reactivation from crude cell extract was measured by a modified method based on the procedures of Ballantine and Boxer [47] and Zhang *et al.* [48]. *E. coli* HypB deletion strain (F^−^, Δ(araD-araB)567, ΔlacZ4787 (::rrnB-3), &lambda^−^, ΔhypB731::kan, rph-1, Δ(rhaD-rhaB)568, hsdR514) was obtained from the Keio collection, *E. coli* Genetic Stock Center [49], and was transformed with the wild-type pBAD-EcHypB or its mutants, pBAD-EcHypB-K224A. The Δ*hypB* genotype and the pBAD-EcHypB plasmid were selected by 50 µg/ml kanamycin and 100 µg/ml ampicillin. Starter culture was grown from single colonies in LB medium at 37°C. After 2% (v/v) inoculation, the *E. coli* cells were grown anaerobically using 50 mL sealed plastic tubes in buffered TGYEP medium (10 g/L tryptone, 5 g/L yeast extract, 0.8% glycerol, 69 mM K_2_HPO_4_, and 22 mM KH_2_PO_4_) supplemented with 15 mM sodium fumarate, 1 µM sodium molybdate, and 1 µM sodium selenite for 16 hours. The expression was induced by adding 1 µM arabinose. Cells were harvested by centrifugation and washed with 50 mM potassium phosphate buffer, pH 7.0, and resuspended in the same buffer containing 1 mM dithiothreitol and 0.2 mM phenylmethylsulfonyl fluoride. Crude cell extracts were prepared by sonication and subsequent centrifugation at 13,000× g for 30 min at 4°C. The protein concentrations of crude cell extracts were determined by the Bradford protein assay (BioRad) using bovine serum albumin as standard.

Total hydrogenase activity of the crude cell extracts was measured by hydrogen-dependent reduction of benzyl viologen [47]. Reactions were prepared in a septum-sealed cuvette. Sealed cuvette containing 2.5 mL of 50 mM potassium phosphate, pH 7.0 was degassed thoroughly by vacuum pump followed by bubbling with 95% N_2_ and 5% H_2_ for 15 minutes. 200 µL solution of 0.05% (w/v) sodium dithiolate was injected into the cuvette to remove the remaining oxidative species. To start a reaction, 300 µL of degassed diluted crude cell extract (at 0.2 mg/mL of total protein) was injected into the cuvette. The initial OD600 should be in the range of 0.2–0.5 to ensure there was no remaining oxidative species in the reaction. OD600 was monitored at 25°C for 15 minutes. The amount of benzyl viologen reduced was quantified by changes in OD600 using the extinction coefficient of 7400 M^−1^. Hydrogenase activity was measured as nanomol of benzyl viologen reduced per min per mg of total protein. Activity measurement of each condition was repeated for three times in individual experiment. Hydrogenase activity of the wild type *hypB* parental strain *E. coli* (F-, Δ(araD-araB)567, ΔlacZ4787(::rrnB-3), &lambda^−^, rph-1, Δ(rhaD-rhaB)568, hsdR514) transformed with pBAD-A was measured along with each batch of reaction as positive control and relative standard.

## Supporting Information

Figure S1 **Switch I region accounts for the major structural change between apo-form HypB and GTPγS-bound HypB.** (A) *A. fulgidus* HypB in apo-form (cyan) (PDB:2WSM) and *M. jannaschii* HypB in GTPγS-bound form (white) (PDB: 2HF8) are superimposable. (B) Cα atom displacement of apo-AfHypB and MjHypB-GTPγS. Major structural difference between the two forms is found in helix 3 and the flanking loops (residue 65–81), where switch I is located. On the contrary, switch II (residue 115–131) shows small structural change. (C) Sequence Logo representation of residues in the switch I region. After removal of 90% redundancy, HypB sequences from 68 species retrieved from NCBI non-redundant database were aligned. The alignment was sent to the WEBLOGO server to create the sequence logo representation. Note that residues Asp-66, Asp-72 and Arg-75 are highly conserved.
(TIF)

Figure S2 **Dissociation constants of AfHypB and the K148A variant for binding guanine nucleotides were determined by titration experiment.** MANT-labeled nucleotide analogues were titrated into AfHypB and the K148A variant. The dissociation constants were determined by fitting the fluorescence emission against the ligand titrated as described in the Methods and Materials.
(TIF)

Figure S3 **GTPase activity of AfhypB was not significantly altered by K148A mutation.** GTPase activity of AfHypB and AfHypB K148A with enzyme-free negative control measured as free phosphate release from GTP in 50 µL reaction mix. (A) Reactions were performed in triplicate with 200 µM HypB or mutant and 2 mM GTP at 37°C. Free phosphate concentration was determined hourly from zero to eight hours. (B) The hydrolysis activity assay was repeated with HypB pre-incubated with equimolar of GDP.
(TIF)

Figure S4 **Binding of guanine nucleotide to HypB requires magnesium.** 1 µM of AfHypB was mixed with 1 µM of MANT-GDP and the fluorescence emission spectrum with excitation 290 nm of AfHypB in complex with MANT-GDP in 50 mM Tris pH 7.5, 0.2 M NaCl, 10 µM MgCl_2_ was measured (black solid line). Fluorescence emission in the range 400–500 nm dropped significantly when 5 mM EDTA was included in the buffer mixture (grey), showing that reducing the availability of magnesium with EDTA inhibit binding of guanine nucleotide to AfHypB. Base fluorescence of MANT-GDP with EDTA and buffer alone was also measured (dashed line).
(TIF)

Figure S5 **Simulated annealing omit map of the crystal structure of AfHypB.** A simulated omit map of the crystal structure of AfHypB was generated. The core region between helix-2, helix-11 and central beta sheet at map contour 1.5 sigma was shown.
(TIF)

Table S1 **DNA primers used to create clones.** The pRSET-His-SUMO vector is a home-made vector by inserting a SUMO tag into pRSET-A (Invitrogen). For restriction enzymes and ligation cloning, the restriction sites in the primer are underlined and the sequences that prime to the hypB sequences encoding the desired HypB proteins are in upper case letters. For Quikchange mutagenesis, the mismatching bases are in lower case letters.
(DOC)
