# Supplementary figures and images for: Structural Basis for GTP-Dependent Dimerization of Hydrogenase Maturation Factor HypB
Source: PLoS One. 2012 Jan 20;7(1):e30547. doi: 10.1371/journal.pone.0030547 (PMC3262836; doi:10.1371/journal.pone.0030547)

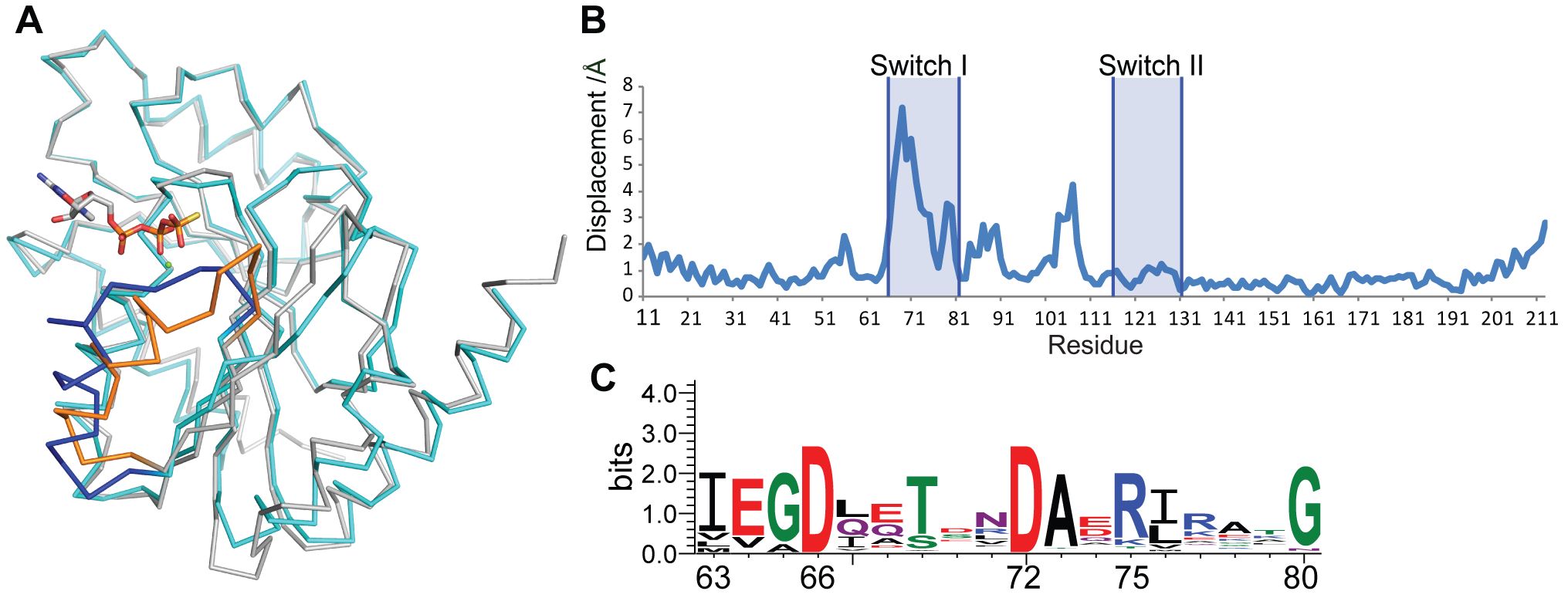

Supplement: Figure S1 — Switch I region accounts for the major structural change between apo-form HypB and GTPγS-bound HypB. (A) A. fulgidus HypB in apo-form (cyan) (PDB:2WSM) and M. jannaschii HypB in GTPγS-bound form (white) (PDB: 2HF8) are superimposable. (B) Cα atom displacement of apo-AfHypB and MjHypB-GTPγS. Major structural difference between the two forms is found in helix 3 and the flanking loops (residue 65–81), where switch I is located. On the contrary, switch II (residue 115–131) shows small structural change. (C) Sequence Logo representation of residues in the switch I region. After removal of 90% redundancy, HypB sequences from 68 species retrieved from NCBI non-redundant database were aligned. The alignment was sent to the WEBLOGO server to create the sequence logo representation. Note that residues Asp-66, Asp-72 and Arg-75 are highly conserved. (TIF) [file pone.0030547.s001.tif]

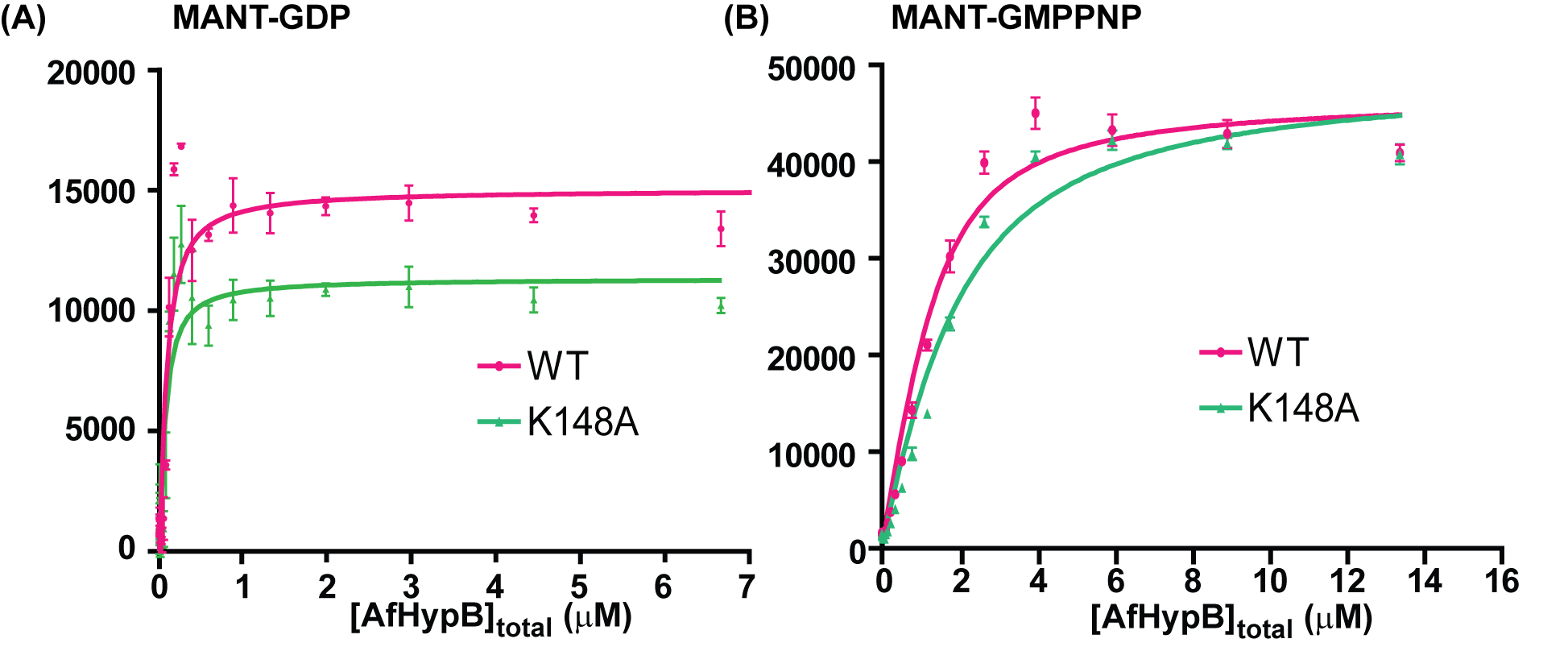

Supplement: Figure S2 — Dissociation constants of AfHypB and the K148A variant for binding guanine nucleotides were determined by titration experiment. MANT-labeled nucleotide analogues were titrated into AfHypB and the K148A variant. The dissociation constants were determined by fitting the fluorescence emission against the ligand titrated as described in the Methods and Materials. (TIF) [file pone.0030547.s002.tif]

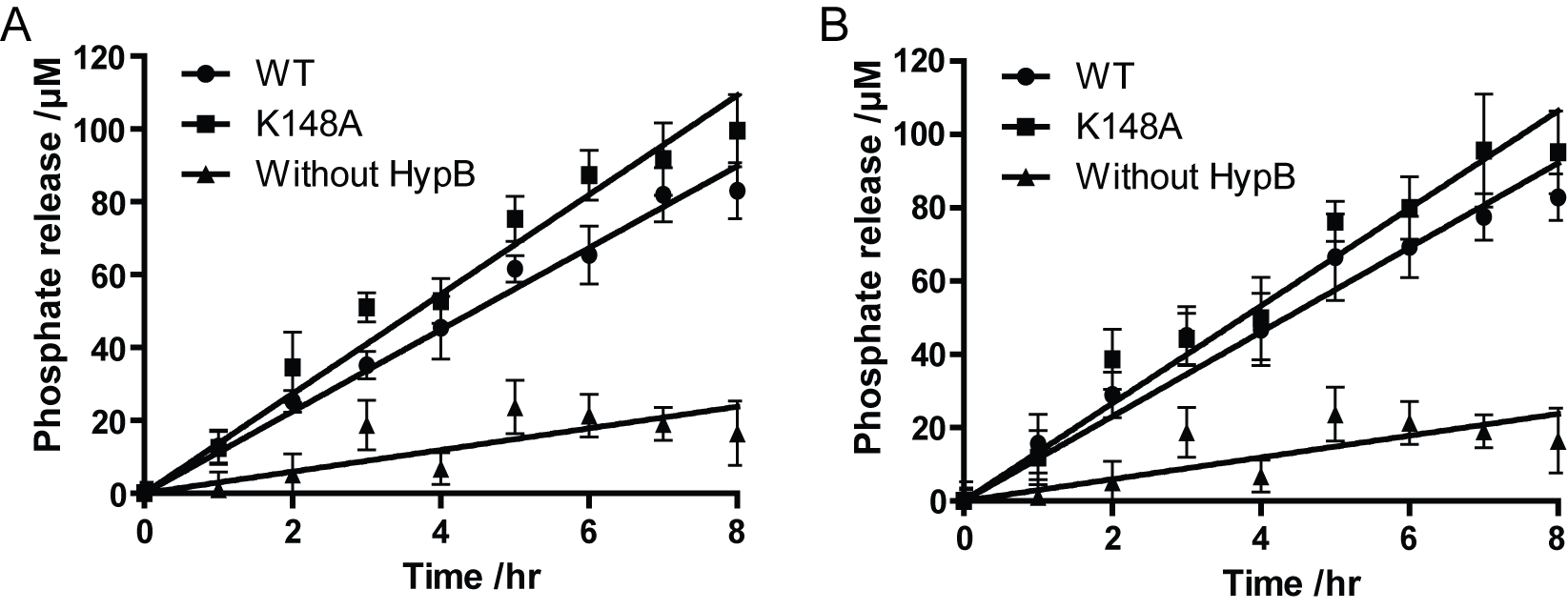

Supplement: Figure S3 — GTPase activity of AfhypB was not significantly altered by K148A mutation. GTPase activity of AfHypB and AfHypB K148A with enzyme-free negative control measured as free phosphate release from GTP in 50 µL reaction mix. (A) Reactions were performed in triplicate with 200 µM HypB or mutant and 2 mM GTP at 37°C. Free phosphate concentration was determined hourly from zero to eight hours. (B) The hydrolysis activity assay was repeated with HypB pre-incubated with equimolar of GDP. (TIF) [file pone.0030547.s003.tif]

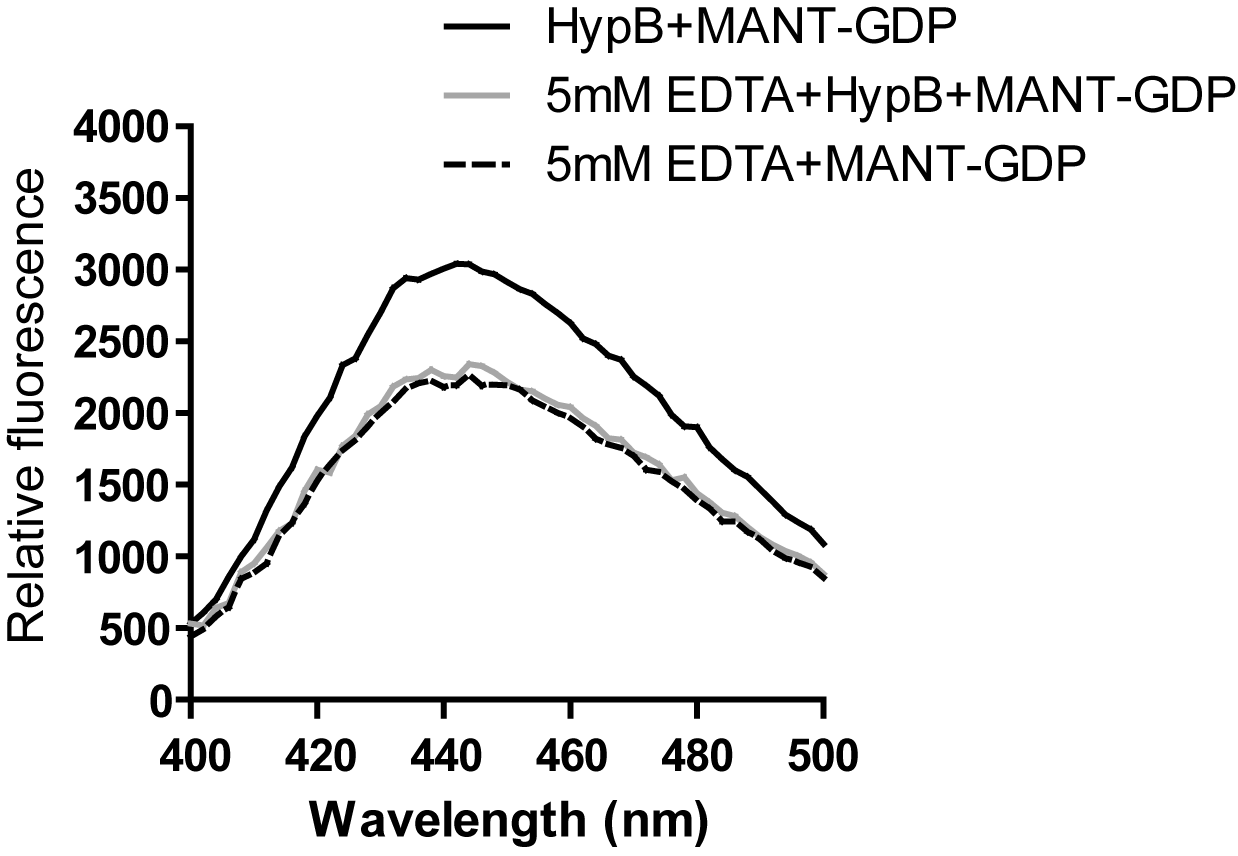

Supplement: Figure S4 — Binding of guanine nucleotide to HypB requires magnesium. 1 µM of AfHypB was mixed with 1 µM of MANT-GDP and the fluorescence emission spectrum with excitation 290 nm of AfHypB in complex with MANT-GDP in 50 mM Tris pH 7.5, 0.2 M NaCl, 10 µM MgCl2 was measured (black solid line). Fluorescence emission in the range 400–500 nm dropped significantly when 5 mM EDTA was included in the buffer mixture (grey), showing that reducing the availability of magnesium with EDTA inhibit binding of guanine nucleotide to AfHypB. Base fluorescence of MANT-GDP with EDTA and buffer alone was also measured (dashed line). (TIF) [file pone.0030547.s004.tif]

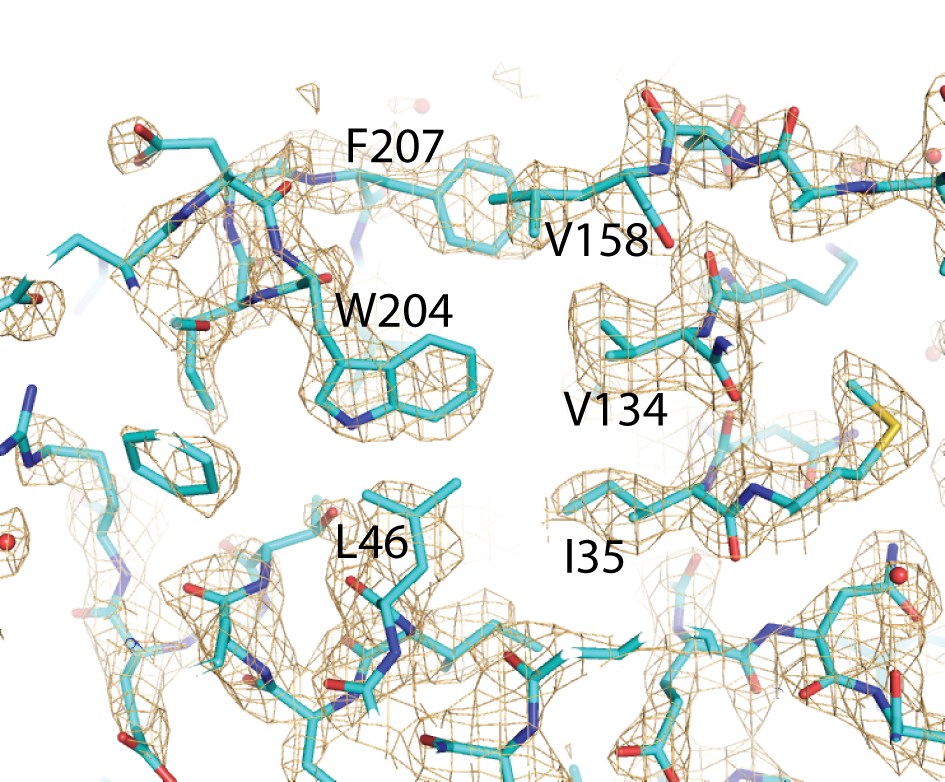

Supplement: Figure S5 — Simulated annealing omit map of the crystal structure of AfHypB. A simulated omit map of the crystal structure of AfHypB was generated. The core region between helix-2, helix-11 and central beta sheet at map contour 1.5 sigma was shown. (TIF) [file pone.0030547.s005.tif]
