# Supplementary material for: Structural Basis for GTP-Dependent Dimerization of Hydrogenase Maturation Factor HypB
Source: PLoS One. 2012 Jan 20;7(1):e30547. doi: 10.1371/journal.pone.0030547 (PMC3262836; doi:10.1371/journal.pone.0030547)
Supplement: Table S1 — DNA primers used to create clones. The pRSET-His-SUMO vector is a home-made vector by inserting a SUMO tag into pRSET-A (Invitrogen). For restriction enzymes and ligation cloning, the restriction sites in the primer are underlined and the sequences that prime to the hypB sequences encoding the desired HypB proteins are in upper case letters. For Quikchange mutagenesis, the mismatching bases are in lower case letters. (DOC) [file pone.0030547.s006.doc]

**Table S1.** Primers used to create clones

| **Constructs** | **Primer used** |
| --- | --- |
| *Restriction enzymes and ligation* |  |
| pRSET-His-SUMO-AfHypB | tatgcaggatccATGCACGAGTATGAACTTAATC  tatgcagaattcTCAATTCTGCCCGGAATC |
| pBAD-EcHypB | tatgcaccatgggtATGTGTACAACATGCGGTTG  tatgcagaattcCTATGCACATCGCTGTGTC |
| *Quikchange mutagenesis* |  |
| pRSET-His-SUMO-AfHypB-K148A | | GATGACGTGGTTGAAgcgCATCCAGAGATTTTC | | --- | | GAAAATCTCTGGATGcgcTTCAACCACGTCATC | |
| pBAD-EcHypB-K224A | | GAAGACAAACCACTGgcgTATCCGCATATGTTTGC | | --- | | GCAAACATATGCGGATAcgcCAGTGGTTTGTCTTC | |
